# On studentized residuals in the quantile regression framework

**DOI:** 10.1186/s40064-016-2898-6

**Published:** 2016-08-02

**Authors:** Edmore Ranganai

**Affiliations:** Department of Statistics, University of South Africa, Roodepoort, South Africa

**Keywords:** Leverage, Outlier, Studentized residual, Regression quantiles, Elemental set, Elemental regression, Elemental predictive residual

## Abstract

Although regression quantiles (RQs) are increasingly becoming popular, they are still playing a second fiddle role to the ordinary least squares estimator like their robust counterparts due to the perceived complexity of the robust statistical methodology. In order to make them attractive to statistical practitioners, an endeavor to studentize robust estimators has been undertaken by some researchers. This paper suggests two versions of RQs studentized residual statistics, namely, internally and externally studentized versions based on the elemental set method. The more preferred externally studentized version is compared to the one based on standardized median absolute deviation (MAD) of residuals using a well-known data set in the literature. While the MAD based outlier diagnostic seemed to be uniform and more aggressive to flagging outliers the RQ externally studentized one exhibited a dynamic pattern consistent with RQ results.

## Background

Tukey (1979) recommends that it is perfectly proper to routinely use both the ordinary least squares (OLS) and robust estimators and only examine the data more closely in case of “large” discrepancies-whatever this means (but it is widely accepted that this means that otherwise it suffices to use the OLS). However, this is rarely done as robust estimators are still playing a second fiddle role to the OLS estimator, despite their proliferation. The main reason why this status quo remains is that at the interface of statistics and its applications there are non-specialists who find it insurmountable to deal with this vague idea of “large” discrepancies and the necessary choices of types of estimators and tuning constants involved in the robust statistical methodology. On the other hand the OLS has a clear and easy to implement methodology to conduct inference and goodness of fit analysis (including residual diagnostics). To make the robust estimators more appealing to statistical practitioners, an endeavor to studentize robust estimators has been undertaken by some researchers (see e.g. Mckean and Sheather 1991; Yohai et al. 1991). This studentization enables users to undertake pertinent statistical tests and obtain confidence intervals and critical values as well as outlier diagnosis which parallel the OLS ones.

Outliers (unusual observations in the *Y*-space) can adversely influence the regression model fit thereby invalidating the pertinent statistical inferences (see e.g. Rousseeuw and Leroy 2003; Barnett and Lewis 1998). The Koenker and Basset (1978) regression quantiles (RQs) are fairly robust to outliers as their influence functions are bounded in the *Y*-space. As a result, not only have RQs been employed as alternatives and complementary tools to the OLS estimator but also in robust outlier detection techniques (Portnoy 1991). These detection methods are based on a two-fold approach, namely, the “peeling” of observations fit exactly by extreme RQs and those based on RQ computation, i.e., observations lying below the RQs hyperplanes ${{\widehat{q}}_{Y|{\mathbf {x}}}}(\tau )$ and/or lying above ${{\widehat{q}}_{Y|{\mathbf {x}}}}(1-\tau )$ corresponding to $\widehat{\varvec{\beta }}(\tau )$ and $\widehat{\varvec{\beta }}(1-\tau )$, $\tau \in (0,1)$, respectively (see expression (7)) may be identified as outliers. Complemented by the ordinary least squares (OLS) one consequence of the latter approach is the Ruppert and Carroll (1980) regression trimmed mean estimator. Outliers in the *X*-space are referred to as high leverage points. A worse outcome can result if outliers are further coupled with high leverage points in a data set than when either data aberration manifests alone, especially in the case of RQs. This stems from the fact that RQs are very susceptible to high leverage points since their influence functions are unbounded in the *X*-space. This curtails their effectiveness to detect outliers that are also high leverage (outlier-leverage) points due to the not yet so well-perceived trade-off between the RQs high affinity for high leverage points and their exclusion of (resistance to) outliers. Studentization may be a solution as it involves incoorperating some *X*-information.

Most of the existing outlier diagnostics in the RQ framework are in relation to the global orientation (centre) of the data and not relative to each quantile level $\tau \in (0,1)$, i.e., a conditional quantile model, ${{Q}_{Y|X}}(\tau )$, especially extreme ones. Very few quantile level specific diagnostics exist. One such single case outlier diagnostic in existence is based on the standardized median absolute deviation (MAD) of residuals (Huber and Ronchetti 2009). Given that it is well-known that regression outlier diagnostics do not always agree in flagging outliers the conventionally agreed practice of employing a wide spectrum of diagnostics before the analyst arrives at a verdict cannot be exercised in the RQ framework. The focus of this paper is to contribute by adding some new outlier diagnostics to the few existing ones in the RQ framework and further bring in the OLS’s attractiveness to this framework via studentization of residual statistics. This is a convenient approach as RQs have a common link with the OLS estimator that can be fruitfully exploited. This link exists via the elemental set (ES) method (Hawkins et al. 1984). So a studentized residual statistics are suggested for RQs here based on the ES method.

An ES consists of exactly the minimum number (*p*) of observations to fit the regression model parameters. Such a proposal is motivated by the fact that the basic optimal solution of a linear programming (LP) problem giving a RQ coincides with the *p* points of an ES (see Koenker and Basset 1978, Theorem 3.1; Ranganai 2016). Applying the OLS procedure to the *p* ES observations yields a specific elemental regression (ER). Thus RQ leverage and residual statistics and ER ones are identical. A deterrent to employing the ES method is the possibly huge load involved in computing all the $$K=\left( {\begin{array}{c}n\\ p\end{array}}\right)$$. However, the number of LP optimization solutions giving RQs is approximately equal to $n<K$. Thus the ES approach benefits from the existence of efficient LP optimization algorithms giving RQs as solutions. Also, it is shown that the suggested RQ studentized residual statistics follow a *t* distribution from which a wide spectrum of cut-off values can be obtained like their OLS based counterparts. These are desirable attributes for the practitioner.

In summary the motivations for the development of studentized outlier diagnostics in RQ frame work, are the following:

- Very few RQ $\tau$ level specific outlier diagnostics with the efficacy to deal with all outlier configurations currently exist in the literature. Therefore the conventionally accepted practice of employing a wide spectrum of diagnostics cannot be carried out in the RQ framework unless more get developed.
- Use of of efficient LP algorithms lessens the possibly huge load involved in computing all the *K* ESs as approximately $n<K$ RQs from the LP solutions are of interest to this study.
- Ease of implementation via OLS and the existence of a wide spectrum of cut-off values from the *t* distribution brings in the attractive of OLS to practitioners.
- There is need to develop more single case outlier diagnostics in light of the not so well perceived opposing phenomena between outlier and high leverage behaviours in outlier-leverage points.
- Outlier-leverage points may be identified better using outlier diagnostics as the suggested studentized diagnostics have some leverage (*X* information) inherent in them unlike the entirely residual (*Y* information) based ones.

Motivated by this background, this paper suggests outlier diagnostics based on studentization and ER. The rest of the paper is organized as follows; Some OLS leverage statistics and residuals are elaborated on in the next section; RQ leverage statistics and residuals are discussed in “Regression quantiles leverage statistics and residuals” section; “Studentized residuals in the quantile regression scenario” section dwells on the construction of the suggested RQ studentized residual statistics; Applications are given in “Applications” section while conclusions are given in the last section.

## Some OLS leverage statistics and residuals

Consider the linear regression model,

1 $$\begin{aligned} {\mathbf{Y}} = {\mathbf{1}}_n \beta _0 + {\mathbf{X}} \varvec{\beta } + \varvec{\varepsilon }, \end{aligned}$$

where ${\mathbf{Y}}$ is an $n\times 1$ vector of response observations, ${\mathbf{1}}_n$ is an $n \times 1$ vector of ones, ${\mathbf{X}}$ is an $n\times (p - 1)$ matrix of predictor variables, $\varvec{\beta }$ is a $(p - 1) \times 1$ vector of regressors, $\varvec{\varepsilon }$ is an $n\times 1$ vector of errors, $\varvec{\varepsilon } \thicksim N_n \left( {\mathbf{0}}_n, \sigma ^2 {\mathbf{I}}_n \right)$, ${\mathbf{0}}_n$ is an $n \times 1$ vector of zeros, and ${\mathbf{I}}_n$ is an $n \times n$ identity matrix. The ${{i}{\text {th}}}$ OLS residual is given by

2 $$\begin{aligned} {{e}_{i}}\,=\,{{Y}_{i}}-{\widetilde{\mathbf {x}}}'_{i}{\widehat{\varvec{\beta }}},\quad 1\le i\le n, \end{aligned}$$

where $\widetilde{{\mathbf{x}}}_i' = \left[ {1}, {{\mathbf{x}}_i'} \right]$ with ${{\mathbf{x}}_i'}$ denoting the ${{i}{\text {th}}}$ row of ${\mathbf{X}}$. It is well-known in the literature that the analysis of (raw) residuals (2) is far less potent in flagging outliers than the analysis of their transformed versions.

There are four versions of transformed residuals most frequently employed to identify outliers in the literature. We list them here in order of increasing effectiveness. These are the normalized, the standardized, the internally studentized and externally studentized residuals. The standardized OLS residuals are given by

3 $$\begin{aligned} {{r}_{i}}\,=\,\frac{{{e}_{i}}}{\widehat{\sigma }},\quad 1\le i\le n, \end{aligned}$$

where $\widehat{\sigma }= \sqrt{MSE}$ with $MSE=SSE/n-p$ and *SSE* denoting the usual OLS sum of squares of the error terms. Substituting $\widehat{\sigma }$ in (3) by $\sqrt{Var({{e}_{i}})}=\widehat{\sigma }\sqrt{ 1-{{h}_{i}}}$ yields the internally studentized residuals

4 $$\begin{aligned} {{t}_{i}}=\frac{{{e}_{i}}}{{{{\widehat{\sigma }}}}\sqrt{1-{{h}_{i}}}},\quad 1\le i\le n, \end{aligned}$$

where ${{h}_{i}}\,=\,\,{{\widetilde{\mathbf {x}}}'_{i}}{{\left( {{{\widetilde{{\mathbf {X}}}'}}_{{}}}{{{\widetilde{\mathbf {X}}}}_{{}}} \right) }^{-1}}{{\widetilde{\mathbf {x}}}_{i}}$, the ${{i}{\text {th}}}$ diagonal element of the hat matrix $\mathbf {H}=\widetilde{\mathbf {X}}{{\left( \widetilde{\mathbf {X}}'\widetilde{\mathbf {X}} \right) }^{-1}}{\widetilde{\mathbf {X}}'}$ denotes the leverage of the ${{i}{\text {th}}}$ observation. Under model (1) assumptions, ${{t}_{i}}$ follows a *t* distribution with $n-p$ degrees of freedom, i.e., ${{t}_{i}} \sim {{t}_{n-p}}$.

Finally, the externally studentized residuals follow from substituting $\widehat{\sigma }$ in (3) by $\sqrt{Var({{e}_{(i)}})}=\widehat{\sigma }_{(i)}\sqrt{(1-{{h}_{i}})}$, where the subscript notation (*i*) indicates the deletion of the ${{i}{\text {th}}}$ observation and ${\widehat{\sigma } }_{(i)}^{2}=[(n-p)\widehat{\sigma }^{2}-{e_{i}^{2}}/{(1-{{h}_{i}})}\;]/[{n-p-1}],$ giving

5 $$\begin{aligned} {{t}_{(i)}}=\frac{{{e}_{i}}}{{{\widehat{\sigma } }_{(i)}}\sqrt{1-{{h}_{i}}}},\quad 1\le i\le n. \end{aligned}$$

Also, like ${{t}_{i}}$, under model (1) assumptions ${{t}_{(i)}}$ follows a *t* distribution with $n-p-1$ degrees of freedom, i.e., ${{t}_{(i)}} \sim {\ }t(n-p-1)$

Another version of the residuals that is often used to assess prediction are the jackknife (predicted) residuals

6 $$\begin{aligned} {{e}_{(i)}}\,\,=\,\,{{Y}_{i}}-{{\widetilde{\mathbf {x}}}'_{i}}{{{\widehat{\varvec{\beta }}}}_{(i)}}=\frac{{{e}_{i}}}{1-{{h}_{i}}},\quad 1\le i\le n. \end{aligned}$$

The jackknife residuals have been found to be more effective than the OLS ones in assessing prediction and flagging outliers in the literature (see e.g. Myers et al. 2010). The predicted sum of squares gives the well-known *PRESS* statistic,

$$\begin{aligned} PRESS_{(i)}= & {} {{\sum \limits _{i=1}^{n}{\left( \frac{{{e}_{i}}}{1-{{h}_{i}}} \right) }}^{2}}. \end{aligned}$$

In the next section some of the analogues of the OLS statistics discussed here are adapted to the RQ scenario.

## Regression quantiles leverage statistics and residuals

The ${{\tau }{th}}$ RQ based on the linear model is a solution to the linear programming (LP) problem

7 $$\begin{aligned} \widehat{\varvec{\beta }}(\tau )\,=\, \underset{\,{{\beta }_{0}},\varvec{\beta }}{\mathop {\arg \text {min} }}\,\sum \limits _{i=1}^{n}{{{\rho }_{\tau }}\left( {{Y}_{i}}-({{\beta }_{0}}+\,{{{{\mathbf {x}}_{i}'}}}\varvec{\beta }) \right) }, \end{aligned}$$

where ${{\rho }_{\tau }}(u)=u[\tau -I(u<0)]\equiv u[\tau \cdot I(u\ge 0)+(\tau -1).I(u<0)]$, for $\tau \in (0,1)$. The basic optimal solution to this LP problem (7) obtained using efficient LP algorithms in the literature, is a RQ that corresponds to a specific ES of size *p* (see Koenker and Basset 1978, Theorem 3.1, p. 39; Koenker 2005, Subsection 2.2.1). Two major linear programming techniques exist for solving the above linear programming problem, viz., exterior and interior methods.

Letting $\widetilde{{\mathbf{X}}} = \left[ {\mathbf{1}}_n, {\mathbf{X}} \right]$ in terms of ESs the linear model (1) can be expressed as

$$\begin{aligned} \left( \begin{array}{c} \displaystyle {\mathbf{Y}}_J \\ \displaystyle {\mathbf{Y}}_I \\ \end{array} \right) = \displaystyle \left( \begin{array}{c} \widetilde{{\mathbf{X}}}_J \\ \displaystyle \widetilde{{\mathbf{X}}}_I \\ \end{array} \right) \varvec{\beta } + \varvec{\varepsilon }, \end{aligned}$$

where $\widetilde{{\mathbf{X}}}_J$ is $p\times p$ and $\widetilde{{\mathbf{X}}}_I$ is $(n-p)\times p$ matrices. Let $$\left( \begin{array}{cc} \widetilde{{\mathbf{X}}}_J&{\mathbf{Y}}_J \end{array} \right)$$ be a generic ES, then $K = {n \atopwithdelims ()p}$ is the number of ESs. The subset *J* corresponds to the set of subscripts $\{{{h}_{1}},...,{{h}_{p}}\}$ such that $({{\mathbf {x}}'_{{{{{hi}}}}}},{{y}_{hi}})$, $i=1,...,p$, is the the ${{i}{\text {th}}}$ case of ES *J*. Applying OLS to an ES based on a subset *J* of size *p* of the original data results in the following vector of regression coefficients estimates

8 $$\begin{aligned} \displaystyle \widehat{\varvec{\beta }}_J = \left( \widetilde{{\mathbf{X}}}'_J \widetilde{{\mathbf{X}}}_J \right) ^{-1} \widetilde{{\mathbf{X}}}'_J {\mathbf{Y}}_J = \widetilde{{\mathbf{X}}}^{-1}_J {\mathbf{Y}}_J, \end{aligned}$$

where $\widetilde{{\mathbf{X}}}_J$ is a square matrix and assumed to be nonsingular. Since a RQ solution of (7) corresponds to ER (8) then their leverage statistics and residuals are identical.

RQ/ER leverage statistics are the diagonal elements of the matrix ${{\mathbf {H}}_{J}}=\widetilde{\mathbf {X}} ({\widetilde{\mathbf {X}}}'_{J}{\widetilde{\mathbf {X}}}_{J})^{-1} \widetilde{\mathbf {X}}'$, i.e.,

9 $$\begin{aligned} h_{iJ}={\left\{ \begin{array}{ll} 1, &\quad \text {for }i\in J\\ \widetilde{\mathbf {x}}_{i}'(\widetilde{\mathbf {X}}_{J}' \widetilde{\mathbf {X}}_{J})^{-1}\widetilde{\mathbf {x}}_{i}, &\quad \text {for }i\not \in J. \end{array}\right. } \end{aligned}$$

The statistic $h_{iJ},\; i \not \in J$ is referred to as the ER predicted (ERP) leverage. Note that this statistic is the jackknife analogue of the ${{i}{\text {th}}}$ diagonal element ${{h}_{(i)}}\,=\,\,{{\widetilde{\mathbf {x}}}'_{i}}{{\left( {{{\widetilde{\mathbf {{X}}'}}}_{(i)}}{{{\widetilde{\mathbf {X}}}}_{(i)}} \right) }^{-1}}{{\widetilde{\mathbf {x}}}_{i}},$ of another variant of the hat matrix ${{\mathbf {H}}_{(i)}}=\widetilde{\mathbf {X}}{{\left( {{{{\widetilde{\mathbf {X}}'}}}_{(i)}}{{{\widetilde{\mathbf {X}}}}_{(i)}} \right) }^{-1}}{\widetilde{\mathbf {X}}'}$.

The RQ/ER residuals are given by

10 $$\begin{aligned} e_{iJ}=\left\{ \begin{array}{ll} 0, &\quad \text {for }i \in J\\ y_{i}-\widetilde{\mathbf {x}}_{i}'\widehat{\varvec{\beta }}_{J}, &\quad \text {for }i \not \in J.\\ \end{array} \right. \end{aligned}$$

The residuals $e_{iJ},\; i \not \in J$ which are the analogues of the jackknife (predicted) residuals (6) are referred to as elemental predicted residuals (EPRs). EPRs have has variance

$$\begin{aligned} Var\left( {{e}_{iJ}} \right) \,\,=\,\,\sigma ^{2}\left( 1+{{h}_{i\,J}} \right) \quad \text {for}\,i\notin J. \end{aligned}$$

Following from this variance, Hawkins et al. (1984) referred to ${{h}_{iJ\,\,}},\,\, i\notin J$ as the residual freedom, to “convey the impression of its property of measuring the extent to which the elemental set *J* fails to predict ${{Y}_{i}}$.” Consequently ${{{e}_{iJ}}}/\sigma {\sqrt{1+hiJ}}\;,\quad i\notin J\sim {\ }N(0,1)$.

Summing the EPRs gives the analogue of the *PRESS* statistic

$$\begin{aligned} PRES{{S}_{J}}=\sum \limits _{i\notin J}{e_{iJ}^{2}}. \end{aligned}$$

Residual analysis in the ER case is redundant, since the ER (internal) residuals suffer from the exact fit property, i.e., the (internal) residuals are constants (zeros), and hence, the same applies for the RQ case. However, the external ones, i.e., ER predicted (ERP) residuals which are the analogues of the jackknife (leave one observation out) residuals are useful. Similarly ERP leverage is also useful. Thus in the next section RQs studentized residuals are constructed using ERP residuals and ERP leverage values.

## Studentized residuals in the quantile regression scenario

In this section we construct a version of studentized residuals for RQs. We do this by first suggesting a scaled version of the RQ predictive residuals (EPRs),

11 $$\begin{aligned} {{t}_{iJ_\tau }}\,\,=\,\frac{{{e}_{iJ_\tau }}}{\widehat{\sigma } _{(J_\tau ) }\sqrt{\left( 1+{{h}_{i\,J_\tau }} \right) }},\quad \text {for}\,i\notin J_\tau \end{aligned}$$

where $J_\tau$ denotes the ES corresponding to the ${{\tau }{th}}$ RQ for $\tau \in (0,1)$ since we are only interested in RQs (ESs corresponding to RQs). The statistic ${{\widehat{\sigma }}_{(J_\tau )}}$ is the scaled prediction variance with the *p* observations left out corresponding to a RQ (ER) $J_\tau$ left out, i.e.

12 $$\begin{aligned} \widehat{\sigma }_{(J_\tau )}^{2}=\frac{PRES{{{{S}'}}_{J_\tau }}}{(n-\alpha )}, \end{aligned}$$

where $PRES{{{S}'}_{J_\tau }}=\sum \nolimits _{i\notin J_\tau }{e_{iJ_\tau }^{2}/(1+{{h}_{iJ_\tau }})\ }$ and $\alpha =2p$ accounting for the *p* parameters as well as the *p* ER observations left out corresponding to ${e}_{iJ_\tau }=0$ for $i\in J_\tau$. In line with the literature convention the RQ externally studentized residuals or externally studentized EPRs (SEPRs) should be based the jackknife residual variance

13 $$\begin{aligned} \widehat{\sigma }_{(i)(J_\tau )}^{2}=\frac{PRES{{{{S}'}}_{(i)J_\tau }}}{(n-\alpha -1)}, \end{aligned}$$

i.e., with the ${{i}{\text {th}}}$ observation left out. This statistic is given by

14 $$\begin{aligned} {{\upsilon }_{(i)J_\tau }}=\frac{{{{\widehat{\varepsilon }}}_{iJ_\tau }}}{\sqrt{\frac{1}{(n-\alpha -1)}PRES{{{{S}'}}_{(i)J_\tau }}}}, \end{aligned}$$

where ${{\widehat{\varepsilon }}_{iJ_\tau }}={{{e}_{iJ_\tau }}}/{\sqrt{1+hiJ_\tau }}\;,\quad i\notin J_\tau$ to flag outliers. The internally studentized version is given by

15 $$\begin{aligned} {{\upsilon }_{iJ_\tau }}=\frac{{{{\widehat{\varepsilon }}}_{iJ_\tau }}}{\sqrt{\frac{1}{(n-\alpha )}PRES{{{{S}'}}_{J_\tau }}}}. \end{aligned}$$

The distributions of the these statistics ((14) and (15)) are given by Theorems 1 and 2 from which we determine the appropriate cut-off values.

### **Theorem 1**

*Under model* (1) t*he RQ externally studentized residuals*${{\upsilon }_{(i)J_\tau }}\sim {\ }t(n-2p-1)$.

### *Proof*

Let $${{\theta }_{i}}={{t}_{iJ_\tau }}\sqrt{1+hiJ_\tau },\quad i\notin J_\tau ,$$ with $${{t}_{iJ_\tau }}=\frac{{{e}_{iJ_\tau }}}{{{{\widehat{\sigma }}}_{(J_\tau )}}\sqrt{1+{{h}_{iJ_\tau }}}}=\frac{{{{\widehat{\varepsilon }}}_{iJ_\tau }}}{{{{\widehat{\sigma }}}_{(J_\tau )}}}.$$

Substituting (12) into ${{\theta }_{i}}$, we have

$$\begin{aligned} {{\theta }_{i}}=\frac{{{{\widehat{\varepsilon }}}_{iJ_\tau }}}{{{{\widehat{\sigma }}}_{(J_\tau )}}}=\frac{{{{\widehat{e}}}_{iJ_\tau }}\sqrt{(n-\alpha )}}{\sqrt{PRES{{{{S}'}}_{J_\tau }}}},\quad i\notin J_\tau . \end{aligned}$$

Therefore

$$\begin{aligned} \theta _{i}^{2}=(n-\alpha )\widehat{\varepsilon }_{iJ_\tau }^{2}/PRES{{{S}'}_{J_\tau }}\ \end{aligned}$$

with ${{\widehat{\varepsilon }}_{iJ_\tau }}$. So $0\le \frac{\widehat{\varepsilon }_{iJ_\tau }^{2}}{PRES{{{{S}'}}_{J_\tau }}}\le 1$ and $\theta _{i}^{2}\le (n-\alpha )$ or equivalently $|{{\theta }_{i}}|\le \sqrt{(n-\alpha )}$ meaning that the density function of ${{\theta }_{i}}$ is zero outside $[-\sqrt{(n-\alpha )},\sqrt{(n-\alpha )}].$ Now let

$$\begin{aligned} {{\upsilon }_{(i)J_\tau }}=\sqrt{\frac{(n-\alpha -1)}{(n-\alpha )(1+{{h}_{iJ_\tau }})}}\frac{{{\theta }_{i}}}{\sqrt{1-\frac{\theta _{i}^{2}}{n-\alpha }}}. \end{aligned}$$

The second factor can be simplified as

$$\begin{aligned} \frac{{{\theta }_{i}}}{\sqrt{1-\frac{\theta _{i}^{2}}{n-\alpha }}}=\frac{\sqrt{(n-\alpha }){{\theta }_{i}}}{\sqrt{n-\alpha -\theta _{i}^{2}}}=\frac{\sqrt{(n-\alpha })\left[ \sqrt{(n-\alpha }){{{\widehat{\varepsilon }}}_{iJ_\tau }}/\sqrt{PRES{{{{S}'}}_{J_\tau }}}\ \right] }{\sqrt{(n-\alpha )-(n-\alpha )\left[ \widehat{\varepsilon }_{iJ_\tau }^{2}/PRES{{{{S}'}}_{J_\tau }}\ \right] }}. \end{aligned}$$

The denominator component in the square root sign can be expressed as

$$\begin{aligned} \frac{(n-\alpha )(PRES{{{{S}'}}_{J_\tau }}-\widehat{\varepsilon }_{iJ_\tau }^{2})}{PRES{{{{S}'}}_{J_\tau }}}=\frac{(n-\alpha )PRES{{{{S}'}}_{(i)J_\tau }}}{PRES{{{{S}'}}_{J_\tau }}}, \end{aligned}$$

where $$PRES{{{S}'}_{(i)J_\tau }}=\sum \nolimits _{j\ne i}{e_{jJ_\tau }^{2}/(1+{{h}_{jJ_\tau }})\ =\sum \nolimits _{j\ne i}{\widehat{\varepsilon }_{jJ_\tau }^{2}\ }},\,\,\text {for}\quad i,j\notin J_\tau .$$ Then

$$\begin{aligned} \frac{{{\theta }_{i}}}{\sqrt{1-\frac{\theta _{i}^{2}}{n-\alpha }}}=\frac{\sqrt{(n-\alpha )}}{\sqrt{PRES{{{{S}'}}_{(i)J_\tau }}}}{{\widehat{\varepsilon }}_{iJ_\tau }}. \end{aligned}$$

Multiplying this result by the first factor in ${{\upsilon }_{iJ_\tau }}$ we have

$$\begin{aligned} {{\upsilon }_{(i)J_\tau }}&=\sqrt{\frac{(n-\alpha -1)}{(n-\alpha )(1+{{h}_{iJ_\tau }})}}\frac{\sqrt{(n-\alpha )}}{\sqrt{PRES{{{{S}'}}_{(i)J_\tau }}}}{{{\widehat{\varepsilon }}}_{iJ_\tau }} \\&=\sqrt{\frac{(n-\alpha -1)}{(1+{{h}_{iJ_\tau }})}}\frac{{{{\widehat{\varepsilon }}}_{iJ_\tau }}}{\sqrt{PRES{{{{S}'}}_{(i)J_\tau }}}}. \end{aligned}$$

Therefore

$$\begin{aligned} {{\upsilon }_{(i)J_\tau }}=\frac{\frac{{{{\widehat{\varepsilon }}}_{iJ_\tau }}}{{{\sigma }}}}{\sqrt{\frac{1}{\sigma ^{2}(n-\alpha -1)}PRES{{{{S}'}}_{(i)J_\tau }}}}=\frac{{{{\widehat{\varepsilon }}}_{iJ_\tau }}}{\sqrt{\frac{1}{(n-\alpha -1)}PRES{{{{S}'}}_{(i)J_\tau }}}}\sim {\ }t(n-\alpha -1) \end{aligned}$$

since $\frac{{{{\widehat{\varepsilon }}}_{iJ_\tau }}}{{{\sigma }}}={{{e}_{iJ_\tau }}}/\sigma {\sqrt{1+hiJ_\tau }}\sim {\ }N(0,1)$ and ${\frac{1}{\sigma ^{2}(n-\alpha -1)}PRES{{{{S}'}}_{(i)J_\tau }}}\sim {\ }{{\chi }^{2}}(n-\alpha -1).$ Taking $\alpha =2p$ we have

$$\begin{aligned} {{\upsilon }_{(i)J_\tau }}\sim {\ }t(n-2p-1). \end{aligned}$$

$$\square$$

### **Theorem 2**

*Under model* (1) *the RQ studentized internally residuals*${{\upsilon }_{iJ_\tau }}\sim {\ }t(n-2p)$.

### *Proof*

The proof follows from that of Theorem 1 by substituting ${(n-\alpha -1)^{-1}}PRES{{{{S}'}}_{(i)J_\tau }}$ with ${(n-\alpha )^{-1}}PRES{{{{S}'}}_{J_\tau }}$ for the estimated EPR variance. Thus the final result becomes

$$\begin{aligned} {{\upsilon }_{iJ_\tau }}=\frac{\frac{{{{\widehat{\varepsilon }}}_{iJ_\tau }}}{{{\sigma }}}}{\sqrt{\frac{1}{\sigma ^{2}(n-\alpha )}PRES{{{{S}'}}_{J_\tau }}}}=\frac{{{{\widehat{\varepsilon }}}_{iJ_\tau }}}{\sqrt{\frac{1}{(n-\alpha )} PRES{{{{S}'}}_{J_\tau }}}}\sim {\ }t(n-\alpha ) \end{aligned}$$

since $\frac{{{{\widehat{\varepsilon }}}_{iJ_\tau }}}{{{\sigma }}}={{{e}_{iJ_\tau }}}/\sigma {\sqrt{1+hiJ_\tau }}\sim {\ }N(0,1)$ and ${\frac{1}{\sigma ^{2}(n-\alpha )}PRES{{{{S}'}}_{J_\tau }}}\sim {\ }{{\chi }^{2}}(n-\alpha ).$ Taking $\alpha =2p$ we have

$$\begin{aligned} {{\upsilon }_{iJ_\tau }}\sim {\ }t(n-2p). \end{aligned}$$

$$\square$$

Therefore the appropriate Bonferroni critical values are $t(1-\alpha /2(n-p);n-2p-1).$ The advantage of these critical values is that the Bonferroni method is simple and allows many comparisons to be made simultaneously while still maintaining an overall confidence coefficient. In the literature externally studentized diagnostics are shown to outperform their internal versions counterparts. Therefore it is preferred here to compare the externally SEPR ${{\upsilon }_{(i)J_\tau }}$’s outlier flagging pattern to the MAD version in the SAS QUANTREG procedure. Using the MAD based version of the RQ predicted residuals, outliers are identified as

16 $$\begin{aligned} {{e}_{iJ_\tau }}\equiv {\left\{ \begin{array}{ll} \text {non}\quad \text {outlier}, &\quad \text {if}\quad {{e}_{iJ_\tau }}\le k{{{\widehat{\sigma }}}_{m}} \\ \text {outlier}, &\quad \text {Otherwise}, \end{array}\right. } \end{aligned}$$

where the multiplier *k* usually takes values, 3, 4 or 5. The scale parameter ${{\widehat{\sigma }}_{m}}$ is the corrected median of absolute values ${{\widehat{\sigma }}_{m}}=\text {median}\left\{ |{{e}_{iJ_\tau }}|/{{\theta }_{0}},1\le i\le n \right\}$, where ${{\theta }_{0}}={{\Phi }^{-1}}(0.75)$ is an adjustment consistency with the normal distribution.

In the next sections the flagging rate of outliers based on this cut-off value in expression (16) and the ones from (14) based on critical values of the *t* distribution are compared using the Hocking and Pendleton (1983) data set.

## Applications

In this Section we consider the Hocking and Pendleton (1983) data set. This data set is a plausible candidate to study the efficacy of the SEPR in flagging outliers as it has various various outlier and high leverage scenarios that are both easy and challenging to deal with in the RQ framework. These include a very high leverage observation 24, an outlier in 17 and two outlier-leverage points 11 and 18 with varying degrees of high leverage. Observation 24 will almost always be included in the ES corresponding to RQs due to RQs affinity for high leverage points. Thus it will often have a zero residual while observation 17 will almost always be excluded in this ES and will often have a very large residual. The challenge is on outlier-leverage points 11 and 18 which will depend on the trade-off of the two antagonistic phenomena, namely, the RQs’ affinity for leverage points versus their exclusion (resistance) to outliers.

**Fig. 1:**
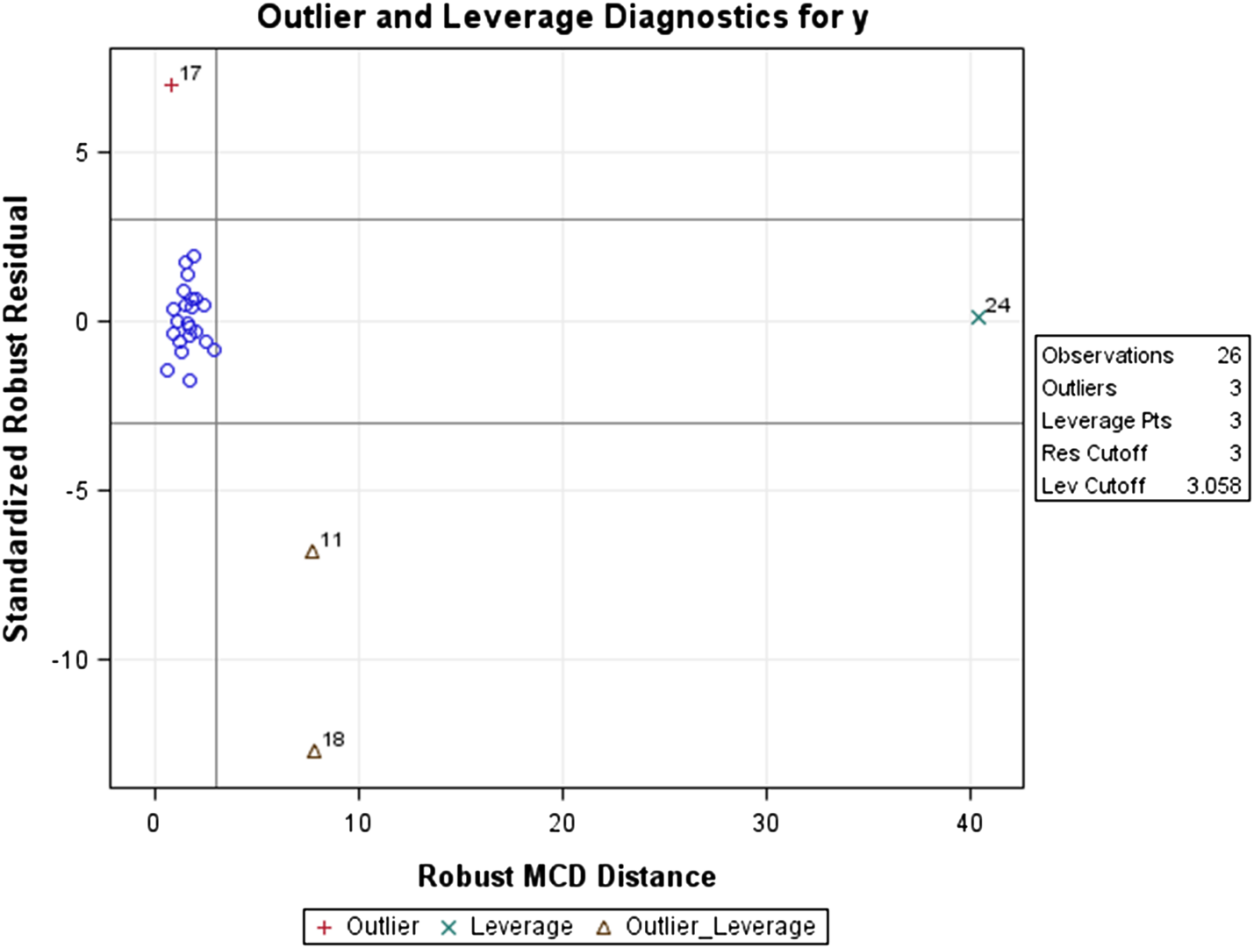
MCD high leverage and outlier diagnosis for the Hocking and Pendleton data set

It is well-known that externally studentized residual statistics always perform better than their internally studentized counterparts since (5) and (14) are based on $\widehat{\sigma }_{(i)}$ and $\widehat{\sigma }_{(i)({{J}_{\tau }})}^{2}$ which are both more robust to problems of gross errors in the ${{i}{th}}$ observation than ${{\widehat{\sigma }}^{2}}$ and $\widehat{\sigma }_{(J_\tau )}^{2}$ on which (4) and (15) are based, respectively (Chatterjee and Hadi 1988, pg 79). Therefore the externally studentized residual criterion (14) is compared to the robust version one based the standardized MAD of residuals (16). Criterion (16) is the only single case similar RQ level related outlier diagnostic with which to validate the efficacy of (14). Firstly the robust and multivariate location and scale diagnostics computed using the minimum covariance determinant (MCD) method of Rousseeuw and Driessen (1999) are applied to circumvent the masking and swamping phenomena so as to expose all the single case high leverage points and outliers. The resulting diagnostic outcome is given in Fig. 1.

The flagging pattern based criteria MAD (16) and SEPR (14) for the Hocking and Pendleton data set are given in Table 1. For criterion (16) the multiplier *k* values were chosen as 3 (*) and 4 (**) while for criterion (14) the significance level $\alpha ={0.10}$ was chosen so as to be both liberal and stringent in flagging outliers. The liberal and stringent Bonferroni cut-off values correspond to $|{{\upsilon }_{(i)J_\tau }}|>t(1-{\alpha }/{2;n-2p-1)}=\pm {1.740}$ and $|{{\upsilon }_{(i)J_\tau }}|>t(1-{\alpha }/{2(n-p);n-2p-1)}\pm {3.544}$, respectively.

**Table 1:** Hocking data set diagnostics

ESs corresponding to RQs				$\tau$	*MAD* (16)	${{\upsilon }_{(i)J_\tau }}$ (14)
8	$11^{\triangle }$	16	$18^{\triangle }$	0.0853	None	$17^+({1.789}^{*})$
8	$11^{\triangle }$	16	19	0.0930	None	$17^+({2.043}^{*})$
8	11	19	$24^{\times }$	0.1232	None	$17^+({2.406}^{*})$
8	12	13	$$24^{\times }$$	0.1861	$$17^+({3.645}^{**})$$, $$18^{\triangle }({-4.582}^{**})$$	$$17^+({1.822}^{*})$$, $$18^{\triangle }({-6.507}^{**})$$
8	13	14	$$24^{\times }$$	0.2046	$$11^{\triangle }({ -4.060}^{**})$$, $$17^+({5.022}^{**})$$, $$18^{\triangle }({-7.486}^{**})$$	$$17^+({2.460}^{*})$$, $$18^{\triangle }({-4.869}^{**})$$
1	14	$$24^{\times }$$	26	0.2528	$$11^{\triangle }({ -4.060}^{**})$$, $$17^+({5.022}^{**})$$, $$18^{\triangle }({-7.486}^{**})$$	$$17^+({2.315}^{*})$$, $$18^{\triangle }({-5.439}^{**})$$
1	5	14	$$24^{\times }$$	0.2593	$$11^{\triangle }({ -4.066}^{**})$$, $$17^+({5.022}^{**})$$, $$18^{\triangle }({-7.494}^{**})$$	$$17^+({2.315}^{*})$$, $$18^{\triangle }({-5.439}^{**})$$
1	14	16	$$24^{\times }$$	0.3053	$$11^{\triangle }({ -5.495}^{**})$$, $$17^+({6.099}^{**})$$, $$18^{\triangle }({-9.149}^{**})$$	$$17^+({1.977}^{*})$$, $$18^{\triangle }({-6.853}^{**})$$
1	4	16	$$24^{\times }$$	0.3659	$$11^{\triangle }({ -6.205}^{**})$$, $$17^+({6.462}^{**})$$, $$18^{\triangle }({-10.647}^{**})$$	$$17^+({2.246}^{*})$$, $$18^{\triangle }({-6.394}^{**})$$
1	14	23	$$24^{\times }$$	0.4018	$$11^{\triangle }({ -6.205}^{**})$$, $$17^+({6.462}^{**})$$, $$18^{\triangle }({-10.647}^{**})$$	$$17^+({2.241}^{*})$$, $$18^{\triangle }({-6.394}^{**})$$
14	16	23	$$24^{\times }$$	0.4412	$$11^{\triangle }({ -6.822}^{**})$$, $$17^+({6.920}^{**})$$, $$18^{\triangle }({-11.740}^{**})$$	$$17^+({1.871}^{*})$$,$$18^{\triangle }({-7.223}^{**})$$
10	14	16	$$24^{\times }$$	0.4686	$$11^{\triangle }({ -6.602}^{**})$$, $$17^+({6.437}^{**})$$, $$18^{\triangle }({-11.162}^{**})$$	$$17^+({2.143}^{*})$$, $$18^{\triangle }({-5.689}^{**})$$
7	10	14	$$24^{\times }$$	0.5370	$$11^{\triangle }({ -6.502}^{**})$$, $$17^+({6.277}^{**})$$, $$18^{\triangle }({-10.923}^{**})$$	$$11^{\triangle }({ -1.741}^{*})$$, $$17^+({2.073}^{*})$$, $$18^{\triangle }({-5.689}^{**})$$
3	9	10	$$24^{\times }$$	0.5448	$$11^{\triangle }({ -6.728}^{**})$$, $$17^+({6.290}^{**})$$,$$18^{\triangle }({-11.073}^{**})$$	$$11^{\triangle }({ -1.741}^{*})$$, $$17^+({2.073}^{*})$$, $$18^{\triangle }({-5.689}^{**})$$
3	8	10	$$24^{\times }$$	0.5512	$$11^{\triangle }({ -6.728}^{**})$$, $$17^+({6.290}^{**})$$, $$18^{\triangle }({-11.073}^{**})$$	$$17^+({1.893}^{*})$$, $$18^{\triangle }({-6.350}^{**})$$
8	9	10	$$24^{\times }$$	0.6215	$$11^{\triangle }({ -7.205}^{**})$$, $$17^+({6.045}^{**})$$, $$18^{\triangle }({-11.492}^{**})$$	$$17^+({2.013}^{*})$$, $$18^{\triangle }({-4.843}^{**})$$
8	9	$$24^{\times }$$	25	0.6315	$$11^{\triangle }({ -7.205}^{**})$$, $$17^+({6.045}^{**})$$, $$18^{\triangle }({-11.492}^{**})$$	$$11^{\triangle }({ -2.301}^{**})$$, $$17^+({2.704}^{**})$$, $$18^{\triangle }({-2.543}^{**})$$
9	15	$$24^{\times }$$	25	0.6839	$$11^{\triangle }({ -7.224}^{**})$$, $$17^+({5.986}^{**})$$, $$18^{\triangle }({-11.488}^{**})$$	$$11^{\triangle }({ -2.132}^{**})$$, $$17^+({2.102}^{**})$$, $$18^{\triangle }({-4.229}^{**})$$
8	9	15	$$24^{\times }$$	0.7227	$$11^{\triangle }({ -7.240}^{**})$$, $$17^+({5.971}^{**})$$, $$18^{\triangle }({-11.476}^{**})$$	$$18^{\triangle }({-6.832}^{**})$$
8	10	15	$$24^{\times }$$	0.7304	$$11^{\triangle }({ -7.240}^{**})$$, $$17^+({5.971}^{**})$$, $$18^{\triangle }({-11.476}^{**})$$	$$18^{\triangle }({-6.832}^{**})$$
6	8	21	$$24^{\times }$$	0.7385	$$11^{\triangle }({ -7.240}^{**})$$, $$17^+({5.971}^{**})$$, $$18^{\triangle }({-11.476}^{**})$$	$$11^{\triangle }({ -1.911}^{*})$$, $$18^{\triangle }({-4.510}^{**})$$
6	21	22	$$24^{\times }$$	0.7660	$$11^{\triangle }({ -6.866}^{**})$$, $$17^+({4.990}^{**})$$, $$18^{\triangle }({-10.409}^{**})$$	$$11^{\triangle }({ -2.236}^{*})$$, $$17^+({2.020}^{*})$$, $$18^{\triangle }({-2.687}^{*})$$
6	8	22	$$24^{\times }$$	0.8276	$$11^{\triangle }({ -5.260}^{**})$$, $$17^+({3.564}^{**})$$, $$18^{\triangle }({-7.887}^{**})$$	$$11^{\triangle }({ -2.807}^{*})$$, $$17^+({1.908}^{*})$$, $$18^{\triangle }({-2.526}^{*})$$
2	6	8	$$24^{\times }$$	0.9549	None	$$11^{\triangle }({ -2.078}^{*})$$, $$18^{\triangle }({-3.067}^{**})$$
6	8	16	$$24^{\times }$$	0.9570	$$11^{\triangle }({ -2.184}^{**})$$, $$18^{\triangle }({-2.897}^{**})$$	$$11^{\triangle }({ -2.078}^{*})$$, $$18^{\triangle }({-3.067}^{**})$$

$+$ Outlier, $\times$ leverage, $\triangle$ outlier-leverage

MAD (16): (*) and (**) corresponds to k=3 and 4, respectively; SEPR (14): (*) and (**) corresponds to *t* values $\pm {1.740}$ and $\pm {3.544}$, respectively

### *Remark*

ESs Corresponding to RQs are the $p=4$ observations (with zero residuals) in the basic optimal solution of LP problem (7) obtained using effeicient linear programing algorithms.

The two outlier diagnostics do not always agree as is the norm in any regression diagnosis outcome using different diagnostics. Observation 24 with the highest leverage and non outlying is never flagged at all. The major difference to note here is the uniform flagging exhibited by (16) from $\tau ={0.2046}$ to $\tau ={0.8276}$ and only otherwise in very extreme $\tau$ levels. It is hard to conceive that results for below and above $\tau ={0.50}$ are similar to this extent. This is inconsistent with the well-known outcome of RQ results due their ability to capture the changing conditional distribution of the response variable, *Y* given the predictor factors, *X* at different quantile levels (Chamberlain 1994; Cade and Noon 2003). On the other hand criterion (14) has a dynamic pattern consistent with RQs results as expected.

## Conclusion

The version of the studentized RQ predicted residuals (SEPRs) suggested here are useful and of benefit to statistical practitioners as they add to the few existing single case outlier diagnostics in the RQ scenario. Further, the methodology is easy to implement as they have cut-off values that parallel the OLS based versions. Thus they offer alternatives to non-specialists who may fight it too hard to comprehend the robust outlier detection methodology. However, if possible these diagnostics must be used together as recommended by Tukey (1979).
